# Totally endoscopic septal myectomy combined with mitral valve replacement for suicide left ventricle following transcatheter aortic valve implantation: a case report

**DOI:** 10.1093/ehjcr/ytag708

**Published:** 2026-09-25

**Authors:** Shogo Maeda, Riku Kato, Toshiaki Ito

**Affiliations:** Department of Cardiovascular Surgery, Japanese Red Cross Nagoya Daiichi Hospital, 3-35 Michishita-cho, Nakamura-ku, Nagoya, Aichi 453-8511, Japan; Department of Cardiovascular Surgery, Japanese Red Cross Nagoya Daiichi Hospital, 3-35 Michishita-cho, Nakamura-ku, Nagoya, Aichi 453-8511, Japan; Department of Cardiovascular Surgery, Japanese Red Cross Nagoya Daiichi Hospital, 3-35 Michishita-cho, Nakamura-ku, Nagoya, Aichi 453-8511, Japan

**Keywords:** Transcatheter aortic valve implantation, Left ventricular outflow tract obstruction, Suicide left ventricle, Totally endoscopic transmitral septal myectomy, Case report

## Abstract

**Background:**

Dynamic left ventricular outflow tract obstruction (LVOTO) causing acute haemodynamic collapse is a rare but life-threatening complication after transcatheter aortic valve implantation (TAVI). Known as suicide left ventricle (SLV), it results from abrupt afterload reduction leading to hypercontractility and dynamic obstruction. Medical therapy is often insufficient, requiring advanced intervention.

**Case summary:**

An 88-year-old woman with symptomatic severe aortic stenosis underwent TAVI. Preprocedural echocardiography showed septal hypertrophy without significant LVOTO. Immediately after valve deployment, she developed LVOTO with systolic anterior motion and severe mitral regurgitation, leading to haemodynamic deterioration. Medical management—including volume loading, discontinuation of inotropes, and beta-blocker—was ineffective. Catheter-based therapies were unsuitable because of severe leaflet calcification. Therefore, totally endoscopic transmitral septal myectomy with mitral valve replacement was performed. Postoperative echocardiography confirmed resolution of LVOTO and mitral regurgitation. She gradually recovered and was transferred for rehabilitation on postoperative Day 132.

**Discussion:**

TAVI can cause SLV in patients with septal hypertrophy. Although transcatheter therapies are generally preferred in patients at high surgical risk, this case demonstrates that totally endoscopic surgery can be a feasible alternative when catheter-based options are not appropriate. Careful preprocedural risk assessment for SLV and consideration of alternative strategies are essential in patients at increased risk of developing this complication.

Learning points

- Careful assessment of the risk of sucide left ventricle (SLV) before transcatheter aortic valve implantation (TAVI) is essential.
- Totally endoscopic transmitral septal myectomy may be a feasible surgical option for selected patients with post-TAVI SLV.
- Alternative treatment strategies should be considered in patients at high risk for SLV.

## Introduction

Dynamic left ventricular outflow tract (LVOT) obstruction (LVOTO) causing acute haemodynamic collapse is a rare but serious complication after transcatheter aortic valve implantation (TAVI). First described by Such *et al*.^1^ in 2010, this condition is known as ‘suicide left ventricle (SLV)’. Among reported cases of SLV presenting with LVOTO or systolic anterior motion (SAM), medical therapy alone was insufficient in 64%, requiring advanced interventions.^2^ These findings emphasize the importance of careful risk assessment before TAVI.

Reported advanced therapies include transcatheter edge-to-edge mitral repair (M-TEER), alcohol septal ablation (ASA), and ventricular pacing^3–5^; however, surgical management has not been described. We report a case successfully treated with totally endoscopic transmitral septal myectomy combined with mitral valve replacement (MVR), achieving a favourable clinical outcome.

## Summary figure

**Figure:**
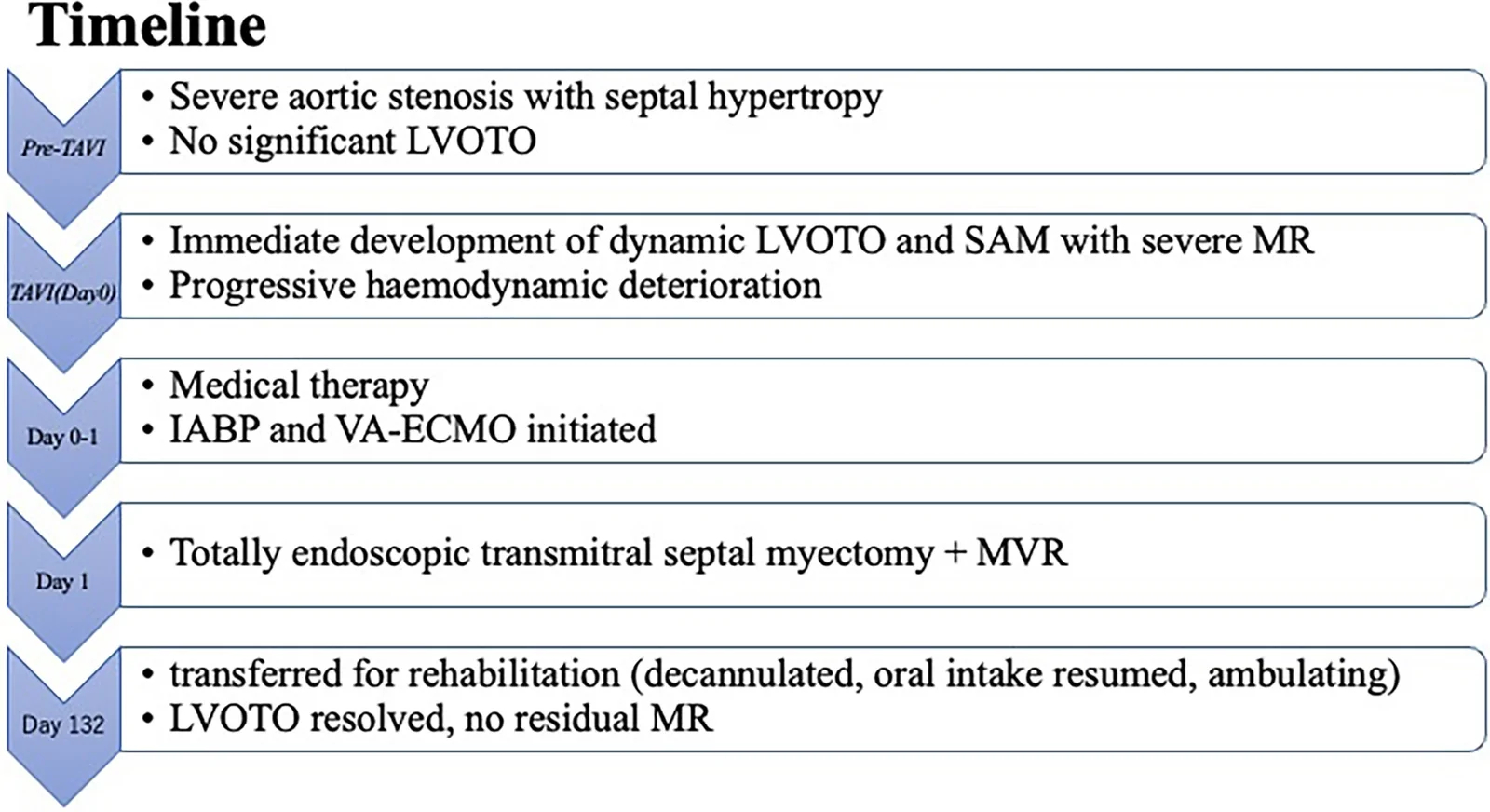


## Case presentation

An 88-year-old woman was diagnosed with severe aortic stenosis during preoperative transthoracic echocardiography prior to rectal cancer surgery. The aortic valve mean gradient was 51 mmHg. Because of symptomatic heart failure, TAVI was selected. Echocardiography showed interventricular septal hypertrophy (17 mm), with a left ventricular end-diastolic diameter of 41 mm and a left ventricular end-systolic diameter of 31 mm. But the LVOT peak gradient was 16.0 mmHg without significant obstruction, and only mild atrial functional mitral regurgitation (MR) was present (*Figure 1*; Supplementary material online, *Videos S1* and *S2*).

**Figure 1:**
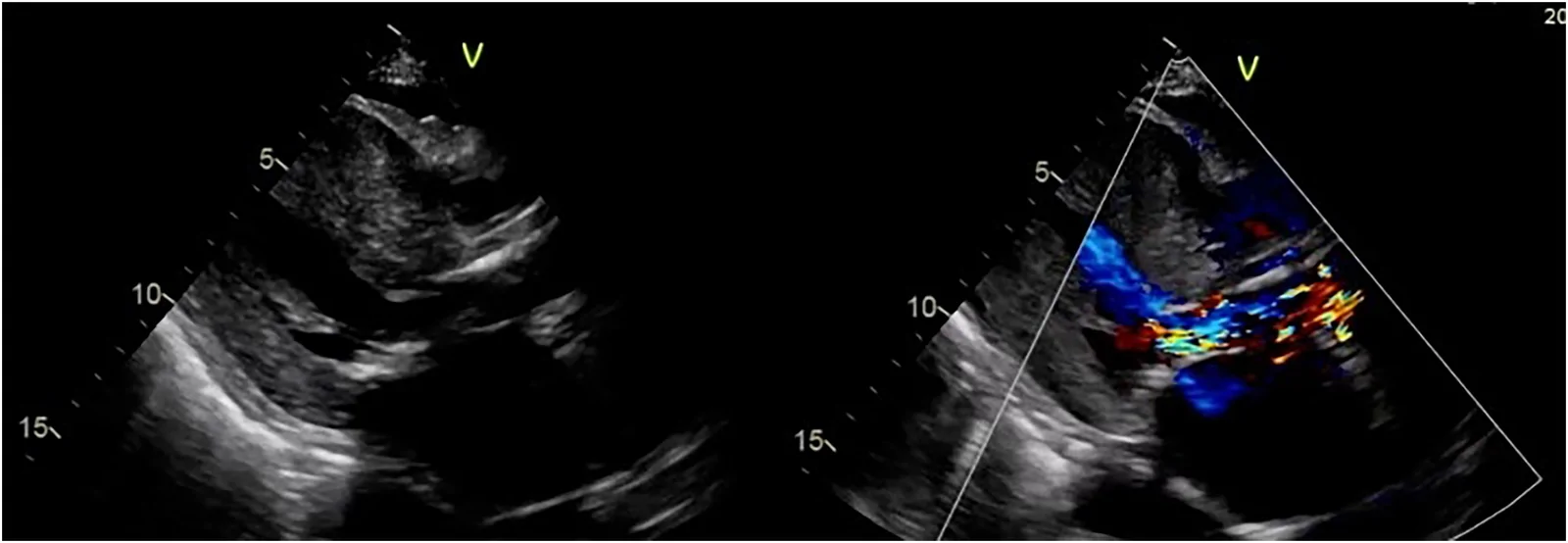
Pre-transcatheter aortic valve implantation transthoracic echocardiography demonstrating severe aortic stenosis with left ventricle hypertrophy and mild atrial functional mitral regurgitation.

Transfemoral TAVI using a 23-mm balloon-expandable valve was performed (*Figure 2A*). Immediately after deployment, the LVOT peak gradient increased to 64 mmHg, indicating dynamic LVOTO, with concomitant SAM and moderate MR (*Figure 2B*; Supplementary material online, *Videos S3* and *S4*). As haemodynamics were initially stable, she was extubated and transferred to the intensive care unit. Her respiratory condition gradually worsened. Medical therapy—including volume loading, withdrawal of catecholamines, and beta-blocker administration—was ineffective, leading to haemodynamic collapse requiring reintubation, intra-aortic balloon pump (IABP) support, and veno-arterial extracorporeal membrane oxygenation (ECMO).

**Figure 2:**
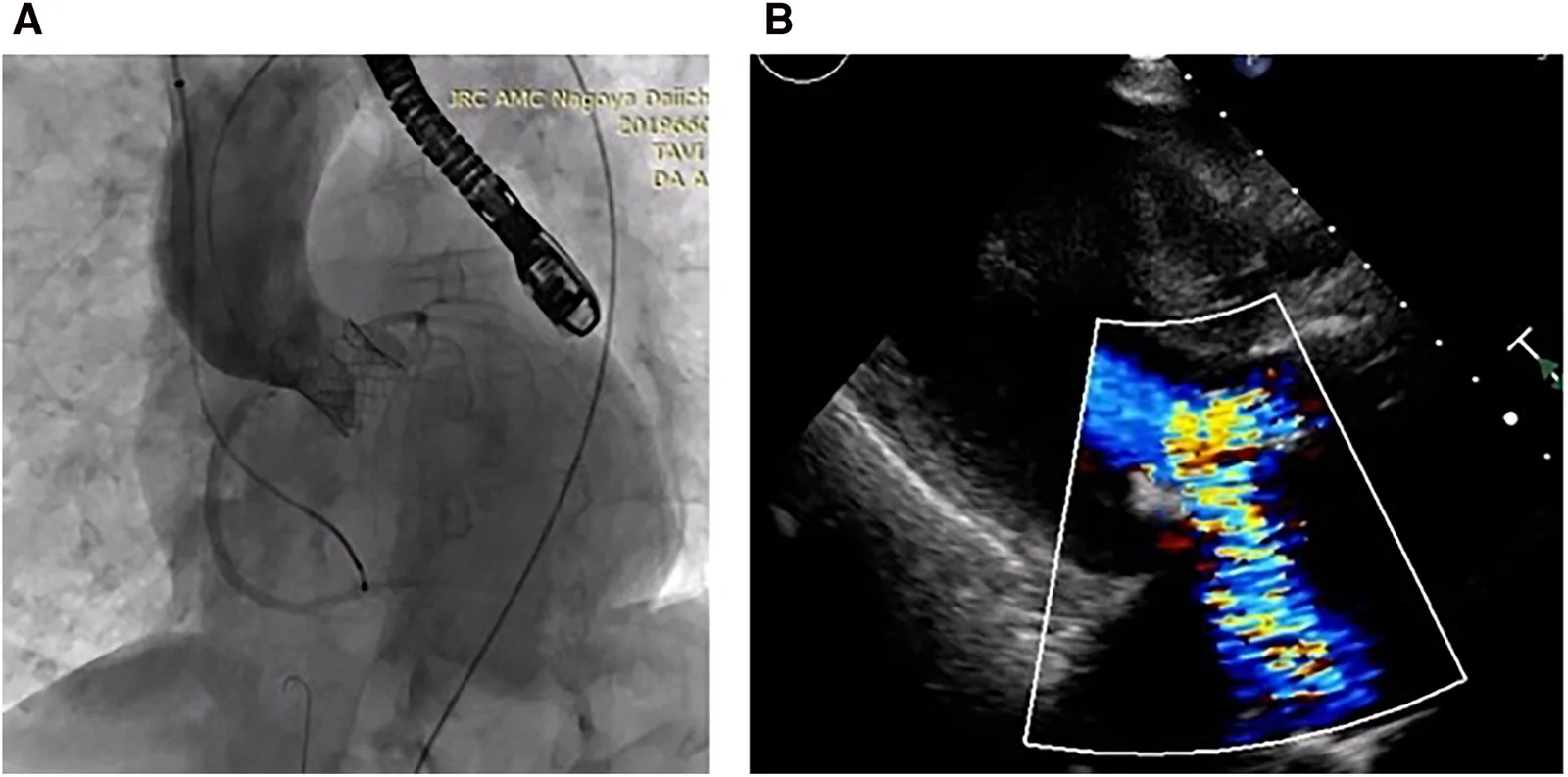
(*A*) Fluoroscopic image immediately after transcatheter aortic valve implantation showing appropriate valve deployment without evidence of paravalvular aortic regurgitation or coronary obstruction. (*B*) Post-transcatheter aortic valve implantation transthoracic echocardiography demonstrating systolic anterior motion associated with severe mitral regurgitation.

M-TEER was considered but deemed unsuitable because of severe leaflet calcification. On the following day, totally endoscopic transmitral septal myectomy with concomitant MVR was performed (Supplementary material online, *Video S5*).

Under a totally endoscopic three-dimensional three-port approach in the left semi-lateral position (*Figure 3*), ECMO was converted to cardiopulmonary bypass. After cardioplegic arrest, the left atrium was opened, revealing leaflet calcification and anterior leaflet atrophy (*Figure 4A*). Following partial preservation of the posterior leaflet, septal myectomy (20 × 30 × 15 mm) was performed below the transcatheter valve to relieve obstruction (*Figure 4B, 4C*), and MVR was completed in a supra-annular position using a 25-mm bioprosthesis (*Figure 4D*).

**Figure 3:**
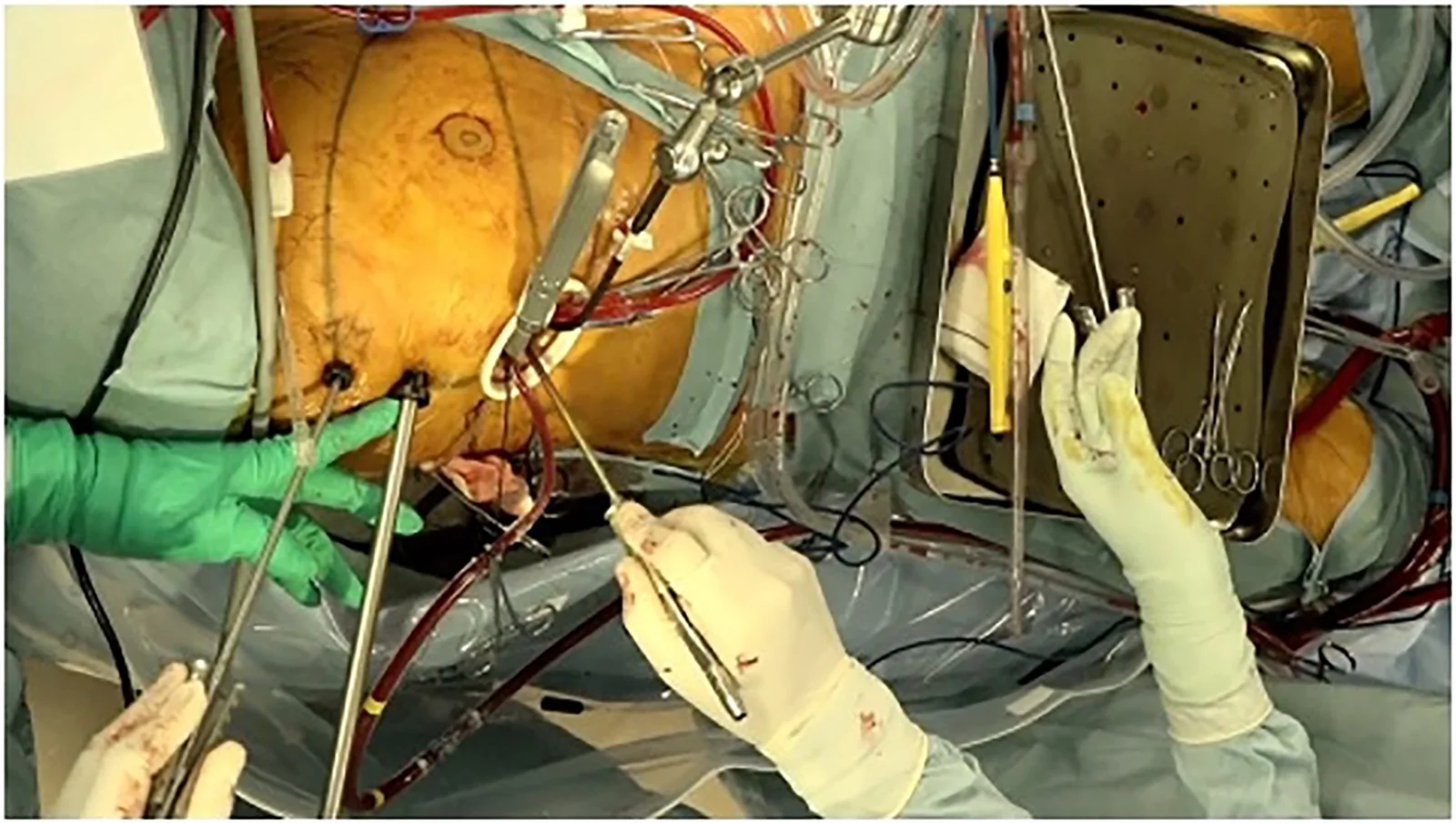
Intraoperative surgical setup using a three-port approach in the left semi-lateral position.

**Figure 4:**
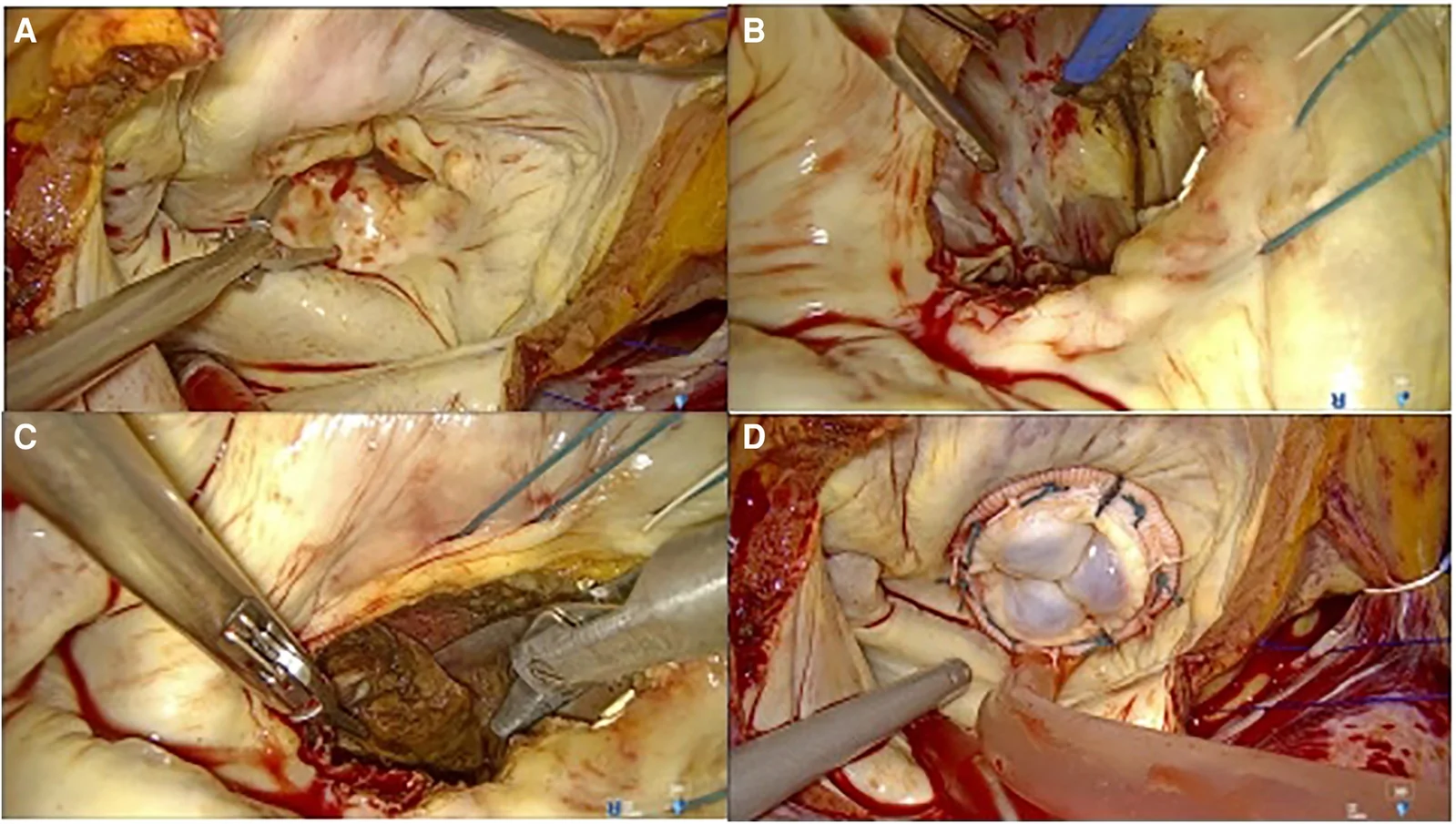
Intraoperative endoscopic findings. (*A*) Severe calcification of the mitral leaflets with anterior leaflet atrophy. Although bulging of the anterior left atrial wall caused by the transcatheter valve is observed, visualization of the mitral valve is adequate. (*B*, C) Transmitral septal myectomy performed after mitral valve resection. A myocardial segment measuring 20 × 30 × 15 mm was resected to relieve left ventricular outflow tract obstruction. (*D*) Implantation of a bioprosthetic mitral valve in the supra-annular position.

Separation from cardiopulmonary bypass was not feasible because of severe preoperative heart failure; ECMO support was therefore resumed. Operative time was 201 min, cardiopulmonary bypass time 117 min, and aortic cross-clamp time 82 min.

ECMO was discontinued on postoperative Day 2, and IABP support was removed on Day 5. Delayed awakening due to cerebral ischaemia from cardiogenic shock necessitated tracheostomy on Day 7. Neurological status gradually improved, enabling decannulation, oral intake, and ambulation. She was transferred for rehabilitation on Day 132. Discharge echocardiography showed resolution of LVOTO (peak gradient 11.6 mmHg) without residual mitral regurgitation (*Figure 5*; Supplementary material online, *Videos S6* and *S7*).

**Figure 5:**
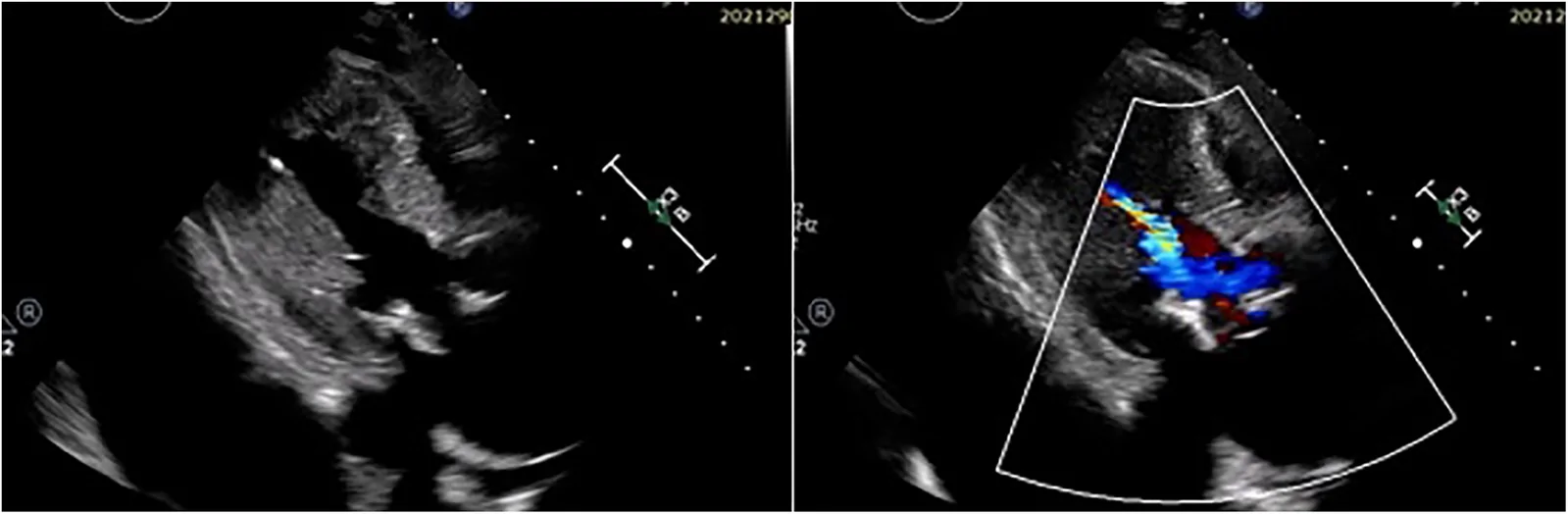
Postoperative transthoracic echocardiography demonstrating complete resolution of left ventricular outflow tract obstruction without residual mitral regurgitation.

## Discussion

In patients with aortic stenosis accompanied by septal hypertrophy, TAVI can precipitate left ventricular hypercontractility and dynamic LVOTO as a consequence of abrupt afterload reduction. This phenomenon, known as ‘suicide left ventricle (SLV)’, occurs in ∼1% of patients undergoing TAVI.^6^ LVOTO is frequently associated with SAM and severe MR, which have been reported in 76% and 67% of patients with SLV, respectively.^2^ Initial management of SLV consists of medical therapy, including volume loading, beta-blockers, discontinuation of inotropes, and phenylephrine. Refractory cases may require more advanced interventions such as ASA, M-TEER, or right ventricular pacing. Although catheter-based advanced therapies for SLV after TAVI have been described, surgical management has not been reported.

In the present case, the appropriateness of an escalated rescue strategy required careful consideration. Although the patient was 88 years old, she had been fully independent in activities of daily living before admission, and the post-TAVI haemodynamic collapse was considered potentially reversible if the mechanical cause of obstruction could be definitively relieved. While prolonged postoperative recovery was anticipated, the immediate prognosis without intervention was poor; therefore, surgical rescue was considered justified despite advanced age.

According to the 2024 AHA guidelines,^7^ ASA is generally preferred over surgical septal myectomy in patients with drug-refractory LVOTO who are at high surgical risk. In this case, TAVI had been selected because of high operative risk, making ASA a reasonable option. However, we judged that ASA would be unlikely to provide immediate haemodynamic stabilization, as it can induce transient septal oedema and delay clinical improvement. M-TEER was also considered but deemed unsuitable due to severe leaflet calcification. Although, in retrospect, surgical aortic valve replacement with concomitant myectomy may also appear reasonable, treatment selection was based on the preprocedural Heart Team assessment. At the time of the initial procedure, the patient’s advanced age and perceived operative risk favoured TAVI.

Given our extensive experience with totally endoscopic mitral surgery and transmitral septal myectomy,^8^ we elected to perform septal myectomy via a totally endoscopic transmitral approach. Compared with median sternotomy, the totally endoscopic strategy is associated with reduced bleeding and facilitates continuation of ECMO support. In addition, the transcatheter aortic prosthesis does not significantly interfere with the operative field through this approach. Although transaortic septal myectomy is the conventional surgical treatment for LVOTO, we selected the transmitral approach because it was less invasive than explanting the TAVI prosthesis and performing surgical aortic valve replacement with concomitant myectomy, and it allowed more reliable control of severe MR. Although MVR eliminates SAM by removing the anterior leaflet, we considered that septal hypertrophy and a small LV cavity could still predispose to residual dynamic LVOTO. Concomitant septal myectomy was therefore performed to enlarge the LVOT and minimize the risk of persistent obstruction after MVR alone. Compared with the conventional transaortic approach, the transmitral endoscopic septal myectomy requires careful definition of the resection plane because of the different operative orientation. In our technique, endoscopic visualization provides excellent exposure of the septum. Using the lookup view, the right coronary cusp is identified as the key anatomical landmark. To minimize the risk of conduction injury, the right margin of the myectomy is kept ∼1 cm to the left of the right fibrous trigone. The superior margin is maintained ∼5 mm below the aortic annulus or, after TAVI, the transcatheter valve skirt. The resection is then extended leftward and towards the papillary muscle base as required to achieve adequate enlargement of the LVOT.^8^ Despite prior TAVI implantation, visualization of both the mitral valve and the septum was excellent, suggesting that this approach may represent a feasible advanced therapeutic option in selected cases when performed in well-experienced institutions with a full available endoscopic surgery programme.

Reported risk factors for SLV after TAVI include female sex, hypertrophic septum, a small left ventricle, and hyperdynamic systolic function.^2,9^ In retrospect, this patient may have been at high risk for SLV. Careful preprocedural assessment of septal morphology, left ventricular cavity size, and the possibility of latent LVOTO may help identify patients at risk for this complication and may influence the choice between TAVI and alternative strategies such as surgical aortic valve replacement with myectomy or staged TAVI after ASA.^10^

## Lead author biography


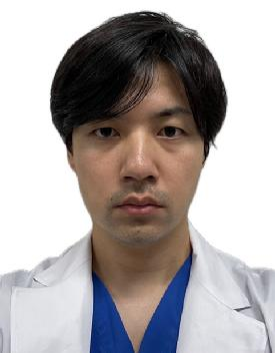
 Dr Shogo Maeda is a cardiovascular surgeon at Japanese Red Cross Nagoya Daiichi Hospital in Nagoya, Japan. His clinical and academic interests focus on minimally invasive cardiac surgery and the management of valvular heart disease. He is particularly committed to advancing less invasive surgical strategies to improve outcomes for patients with valvular disorders.

## Supplementary Material

ytag708_Supplementary_Data

## Data Availability

The data underlying this article are available in the article and in its online supplementary material.

## Supplementary material

**Consent:** This study was approved by the Institutional Review Board of the Japanese Red Cross Nagoya Daiichi Hospital (Approval number: 2015-067; Approval date: 10 June 2015). We have obtained written informed consent from the patient.

## References

[1] Suh WM, Witzke CF, Palacios IF. Suicide left ventricle following transcatheter aortic valve implantation. Catheter Cardiovasc Interv 2010;76:616–620. 10.1002/ccd.22609 20506145

[2] Barzallo D, Torrado J, Benites-Moya CJ, Sturla M, Echarte-Morales J, Scotti A, et al Acute hemodynamic compromise after transcatheter aortic valve replacement due to dynamic left ventricle obstruction: a systematic review. Am J Cardiol 2024;214:125–135. 10.1016/j.amjcard.2023.12.005 38103763

[3] Qintar M, Villablanca P, Lee J, Wang DD, Frisoli T, O'Neill B, et al Emergency alcohol septal ablation for shock after TAVR: one more option in the toolbox. JACC Case Rep 2021;3:853–858. 10.1016/j.jaccas.2021.03.028 PMC8311368 34317641

[4] Grasso C, Scandura S, Buccheri S, Barbanti M, Di Salvo ME, Mangiafico S, et al MitraClip implantation for the treatment of new-onset systolic anterior motion of the mitral valve after transcatheter aortic valve replacement. Ann Thorac Surg 2016;102:e517–e519. 10.1016/j.athoracsur.2016.05.032 27847070

[5] Endo N, Otsuki H, Domoto S, Yamaguchi J. Haemodynamic collapse immediately after transcatheter aortic valve implantation due to dynamic intraventricular gradient: a case report and review of the literature. Eur Heart J Case Rep 2021;5:ytaa565. 10.1093/ehjcr/ytaa565 PMC7873795 33598628

[6] Kobayashi Y, Enta Y, Nakashima M, Saigan M, Satomi N, Teng Y, et al Incidence, predictors, and prognostic impact of left ventricular outflow tract obstruction following transcatheter aortic valve replacement. CJC Open 2025;7:1236–1243. 10.1016/j.cjco.2025.05.020 PMC12457202 41001271

[7] Ommen SR, Ho CY, Asif IM, Balaji S, Burke MA, Day SM, et al 2024 AHA/ACC/AMSSM/HRS/PACES/SCMR Guideline for the management of hypertrophic cardiomyopathy: a report of the American Heart Association/American College of Cardiology Joint Committee on Clinical Practice Guidelines. Circulation 2024;149:e1239–e1311. 10.1161/CIR.0000000000001250 38718139

[8] Ito T, Sawaki S, Tokoro M, Kato R. Longitudinal valvotomy of anterior leaflet for endoscopic transmitral myectomy for hypertrophic obstructive cardiomyopathy. Multimed Man Cardiothorac Surg 2024;2024. doi:10.1510/mmcts.2024.047. 39212331

[9] Romano S, D'Andrea E, Cozac DA, Savo MT, Cecchetto A, Baritussio A, et al Silent threats of the heart: a case series and narrative review on suicide left ventricle post-aortic valve replacement in patients with dynamic LVOT obstruction and aortic stenosis. J Clin Med 2024;13:5555. 10.3390/jcm13185555 PMC11432347 39337045

[10] Ben-Dor I, Rogers T, Medranda GA, Medvedofsky D, Weissman G, Case BC, et al A word of caution before treating aortic stenosis in patients with concomitant LVOT obstruction. JACC Case Rep 2022;4:1162–1168. 10.1016/j.jaccas.2022.01.025 PMC9537090 36213885

